# Lessons Learned from a Two-Round Delphi-based Scenario Study

**DOI:** 10.1016/j.mex.2020.101179

**Published:** 2020-12-13

**Authors:** Ulrike Schmalz, Stefan Spinler, Jürgen Ringbeck

**Affiliations:** a Bauhaus Luftfahrt e.V., Willy-Messerschmitt-Strasse 1, 82024 Taufkirchen, Germany; b WHU – Otto-Beisheim School of Management, Burgplatz 2, 56179 Vallendar, Germany

**Keywords:** Consensus, Delphi method, Expert knowledge, Foresight, Forecasting, Hierarchical cluster analysis, Judgement, Scenario development

## Abstract

The Delphi technique is a suitable methodology for structuring group communication to answer current and prospective research questions within several rounds. The method is used in many disciplines and characterized by anonymity, iteration, controlled feedback, and statistical “group response” (Rowe & Wright, 2001). This technical paper presents practical details and lessons learned from a two-round Delphi-based scenario study in which projections (Delphi statements, questions or hypotheses) were developed with findings from expert interviews and an expert workshop. This Delphi study provides answers to future-related questions for which other research methods are inappropriate. This is depicted as a five-step process, making it easy to follow and replicable, for example to help first-time Delphi-method researchers. In doing so, the authors aim to provide the community with valuable technical insights and guidance for studies applying the Delphi technique both to prospective questions and in other research settings.

• Conducting a Delphi study can be a slow process with respect to receiving feedback from the panelists. Planning an appropriate period for distributing the questionnaire may produce a higher return rate. A sufficient time buffer should be incorporated into project planning.
• Projections that create dissent among the panelists may provide valuable results.
• Data analytics, software programs and online social networks can support researchers, save time and resources, and provide further insights in the process of conducting a Delphi study.

## Graphical abstract

**Figure:**
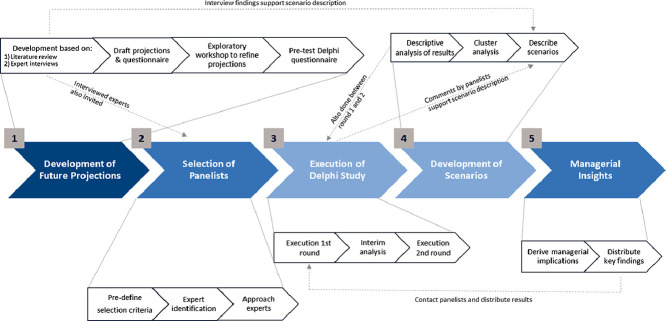

Specifications table

**Table:** 

Subject Area:	Economics and Finance
More specific subject area:	*Future research*
Method name:	*Delphi technique*
Name and reference of original method:	*Dalkey, N., & Helmer, O. (1963). An experimental application of the Delphi method to the use of experts. Management Science, 9(3), 458–467.*
Resource availability:	• Microsoft Office (Word, Excel) • Statistical software (e.g. R or STATA) • Software supporting qualitative data analysis (e.g. NVivo, Atlas.ti) • Web-based platform for RT Delphi (e.g. Calibrum or Mesydel) (Aengenheyster et al. [1] provide a comparison of tools)

## Method details and co-submitted research

### Overview of Delphi method

The Delphi technique, first proposed in the 1950s, was developed by the RAND company [12]. It is an anonymous survey technique conducted in several rounds for structuring group communication. Panel participants are asked to assess projections (also called Delphi statements, questions or hypotheses) repeatedly [29], [30]. The Delphi method has four key characteristics: anonymity, iteration, controlled feedback, and statistical “group response” [43]. Beyond the classical Delphi approach [12], different types exist, such as the nominal group technique, decision Delphi, policy Delphi, and the argument Delphi [20], [38]. It is a suitable technique for answering prospective research questions of what the future might look like (as done in this study) and for instance to assess the expected probability, desirability and expected impact of projections or scenarios. The technique can also focus on current challenges, such as be used to rank and prioritize topics, support policy making, generating ideas, establishing facts, and for other research purposes. Items can also include an assessment on the feasibility, urgency for action, or innovativeness. It is applied mainly in the fields of healthcare, education and business research, with a significant increase in Delphi studies since 2005 [15]. An overview of the most cited papers across the disciplines is provided in the supplementary material (Table 2). Depending on the study type, the Delphi method follows a qualitative, quantitative or mixed-methods approach [38].

The technique can be combined with other methods, for instance to complement the development of projections or to support the analysis of results. There are also differences in the optimal number of panelists, number of rounds, consensus measurement, types of feedback loop and other parameters, giving rise to numerous modified Delphi versions [38], [54] that we believe may be overwhelming for researchers. Over the last decade, various papers have discussed the conduct of Delphi studies from a technical point of view to support researchers. Some focus on specific research steps. An overview of technical papers is provided in the supplementary material (Table 3). Most “lessons learned” papers were published around 2011, and fewer have been published in the last five years [5], [19], [21]. Others focus on specific steps in the process, such as the panelists’ selection [35], or the Delphi questionnaire [33]. However, much has changed in recent years, with modifications to the Delphi technique, including improvements to data analytics, the application of classic theories to development of projections, and tools to support qualitative text analysis of panelists’ comments.

We consider the Delphi technique to be applicable to a wide range of disciplines and a suitable method for experienced, early-career and postgraduate researchers. This technical paper provides additional practical insights for the research community, and complements the literature by providing (1) a discussion of lessons learned from conducting a two-round Delphi study, with practical, step-by-step guidance, and (2) a replicable approach for scholars, including those incorporating scenario developments into their research. We focus on issues regarding the acquisition of experts and panelists, the literature review, development of the Delphi questionnaire, execution of the study, scenario development, and managerial implications for creating practical relevance. We also describe from our own experience and lessons learned on how online social networks, data analytics and research tools such as NVivo can support researchers conducting Delphi studies. We suggest what we would do differently if we were to conduct this or another Delphi study again.

### Co-submitted research

This technical paper is based on a Delphi-based scenario study examining the future of door-to-door (D2D) passenger air travel in 2035 [27]. Today's ever-changing and connected world, with increasingly fragmented customer requirements, also affects the travel industry. Understanding future developments, passenger needs and possible scenarios is crucial for long-term planning and decision making in the mobility sector. In the context of this Delphi study, intermodal D2D air travel is concerned with the entire travel chain, from the point of origin to the final destination. This definition includes not only the air travel segment, but also all access and egress modes to and from the airport, as well as transfers within the airport. Hence, D2D air travel is of high practical relevance to airlines, airports, public transport providers and others. The D2D view on air travel is novel, as many modes have tended to focus only on their own segment or considered D2D travel purely in the context of urban mobility. Applying the D2D view may help to improve the entire travel chain for air travel passengers, for example by creating seamless, intermodal travel or managing disruptions. Our study is particularly concerned with future projections that affect all (or most) aspects of the travel chain, such as digitalization and personalization. However, little is known about what the future might look like in this context. Applying the Delphi technique helps shed some light on possible future D2D air travel scenarios, and enables the inclusion of multi-stakeholder panelists, encompassing the desired scope of multimodality.

### Aim and structure of this technical paper

**Fig. 1:**
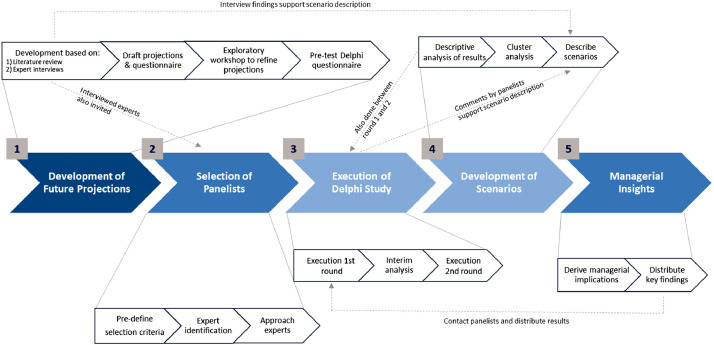
Five-step research approach for a two-round Delphi-based scenario study (adapted from [56] and [27]).

Kluge et al.’s [27] co-submitted study followed a five-step research approach:^1^ 1) development of future projections, 2) selection of panelists, 3) execution of the Delphi study, 4) development of scenarios, and 5) managerial insights (see Fig. 1). This paper is structured according to these five steps, systematically describing the entire research process with technical details. As depicted in Fig. 1, the research steps are interrelated. For instance, findings from the expert interviews in step one are also used at a later stage for the description of scenarios in step four. At the end of each sub-section, we summarize our “*lessons learned”*, in which we present our personal experiences, and aspects we could have improved with hindsight. In doing so, we aim to provide helpful practical recommendations for planning and conducting a Delphi-based scenario study.

## Development of future projections (step one)

The first step involved developing future projections for testing with the Delphi questionnaire, using three qualitative methods: 1) a literature review, 2) expert interviews, and 3) one workshop. Drawing on various sources to develop projections enabled triangulation of methods and ensured that no key topics relevant to the field of study were omitted from the projections. Including industry representatives in the interviews aimed to gain a practical view of the studied topic.

### Literature review

The literature review provided a theoretical background for the projections assessed in the Delphi study. Hence, we consider this step to be indispensable in a Delphi study. We applied Webster and Watson's [58] recommendations for conducting literature reviews. We aimed to examine the latest academic papers on trends around general mobility, D2D travel and air travel, focusing on two key questions: *which trends will influence current and future travel in the D2D context*; and *how will travel behavior and customer needs change?* Using predefined keywords (further developed during the review), we searched the Google Scholar, EBSCO, ABI/INFORM and Scopus databases. We focused on the European market, on leading journals and conference proceedings, and on work published within the last eight years (2010–2018). During the review, we were able to detect high-level trends across studies, such as “digitalization and mobile communications”, “demographic changes” and “gender differences”. These formed the basis for developing the projections.

We also conducted a second literature review of previous Delphi studies on the future of mobility and transport. We identified eight papers and studied their methodological approach and research results. This was helpful for improving our knowledge of applications of the Delphi technique in mobility research, and for gaining insights into potential mobility scenarios and trends. We also created an overview table of previous work, listing author(s) and year, the scope of each study, time horizons, numbers of projections, panelists and rounds, response rates and details of the research. This table was included in the co-submitted paper to provide readers with a systematic summary of previous work.

### Expert interviews

**Fig. 2:**
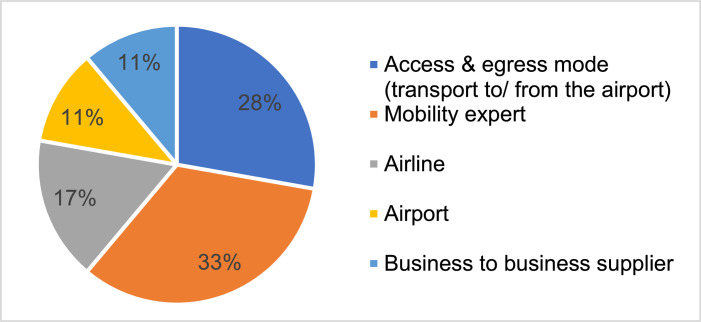
Interviews: distribution of sample (adapted from [41]).

We used interviews in our Delphi study to identify future D2D travel trends and to gain insights into potential future mobility in Europe. The interviews also helped us to develop projections connected with real-world challenges and opportunities in the mobility sector. We designed an interview guide and conducted a test interview with two mobility researchers, as recommended in the literature [8]. After testing and receiving feedback from the test interviewees, the guide was adapted to reduce its length and clarify some wording. The interviews were exploratory in nature, and the guide was used to support a natural conversation rather than creating an interviewee–interviewer situation. We expected that this would uncover topics that we had not previously considered. The final interview guide and the main objectives are presented in the supplementary material. We initially contacted 55 experts via email, 18^2^ of whom agreed to an interview (33% response rate). Our email request incorporated the objective of the interview, an introduction to our research facility, contact details, and information on data usage and storage. As the interviews were part of an EU-project,^3^ we also included details of the project. We conducted 18 semi-structured interviews with experts from industry, such as airlines, airports and public transport providers, and with other mobility experts who were familiar with the topic of D2D air travel. Fig. 2 illustrates the distribution of the sample. As the scope of our research was D2D air travel, it was essential to interview representatives of different modes of transport to create this D2D view. Our focus was on the European market, so we approached experts from various European countries, including Austria (*N* = 1), Belgium (*N* = 1), Switzerland (*N* = 1), Italy (*N* = 1), the Netherlands (*N* = 1) and Germany (*N* = 13).

The interviews were conducted face-to-face or by phone. After obtaining consent, they were recorded and transcribed following Kuckartz's [28] rules for transcription, which focus on content rather than subjective views or perceptions. Analysis of the interview data was based on Mayring's [36] summarizing and inductive category formation approach. We used Microsoft Excel, which can be time-consuming. Findings from the interviews were used to develop a draft list of future projects and to develop the scenarios in research step four (see Section "Development of scenarios (step four)"). All 18 experts interviewed were invited to take part in the Delphi rounds, but only six did so, which may indicate low involvement by some interviewees, although some were constrained by holidays and lack of time.

### Future projections

Having consolidated our findings from the literature review and interviews, we developed key points and 51 draft projections for the Delphi questionnaire. Pre-tests can help to improve the comprehensibility and reliability of Delphi questionnaires. A workshop was conducted with two mobility researchers to discuss and revise the first list of projections. Delphi panelists should not be required to invest too much time in each round, as this may increase the dropout rate. In our study, a list of 17 projections proved to be sufficient. As a result of written pre-tests with two further mobility researchers (neither of whom participated in the interviews or the Delphi questionnaire), a seven-point-Likert ordinal scale was used, as this scale seemed to provide an adequate answer range. Along with the questionnaire, it is also advisable to provide a cover letter, contact details, and a scenario with GDP growth rates and other assumptions. In the questionnaire, the projections were assessed for probability (P), impact (I) and desirability (D). These assessment scales made most sense for our study as we focused on a practical question and the industry impact and desirability were of high interest. Additional scales would have increased the questionnaire length and potentially result into higher dropout rates. The same scales (P, I, & D) were also used in Delphi studies with a transportation scope [17], [47]. Panelists were able to leave short comments on their evaluations (optional). We tried to use simple wording, and provided examples when necessary. Each projection started with “In 2035 …” to remind panelists of the year in scope. We used Microsoft Word to develop the questionnaire in the first and second rounds. An extract from the first-round questionnaire is provided in Fig. 6 in the supplementary material.

One aim of the Delphi method is to shed light on prospective research questions by casting uncertainty in a few concrete scenarios. The year 2035, which was 17 years away in 2018 when our study was conducted, was selected as an appropriate time horizon for two reasons. First, 2035 was imaginable for the experts to make assessments, but sufficiently different from contemporary travel. Second, the mobility sector conducts long-term planning, so projects in around 17 years’ time might provide sufficient time to implement managerial recommendations. More distant time points, such as 2050, would not have fulfilled these two conditions. The literature review also revealed that such time frame was commonly used in other transport-related Delphi studies (e.g. [47], [48], [56]).

### Lessons learned from developing future projections (step one)

• The literature review could have been more systematic. Weißer et al. [59] leverage the power of data analytics (topic filtering and clustering) to identify key topics in a large amount of textual data. This approach may support the detection of relevant trends and preselection of papers for further review, and has gained momentum in recent years. We recommend that researchers should consider data analytics in order to save time and resources, and analyze the literature systematically.
• Our literature review provided the theoretical framework for our Delphi study. Other Delphi studies rely on the application of a theory, such as the diffusion of eco-innovations theory [3] or the organizational information processing theory [42]. In retrospect, we should have used an underlying theory next to the literature review to increase our study results further.
• The use of workshops in the research design could have been expended. As done by Spickermann et al. [48], we could have conducted more workshops to refine projections further but also to derive stakeholder recommendations in step five. Workshops can be conducted with researchers but also with panelists. Saving resources, workshops could also be conducted online.
• It can be time-consuming to get confirmations for interviews. Friendly reminders and (multiple) follow-up emails may help secure positive replies. Our personal networks, and even very loose ties, also helped to bring experts on board. On the other hand, leveraging personal networks, sometimes known as *friendomization*, may create biases. One bias may have been the geographical distribution of our sample, as explored below. Incentives can also be helpful, as discussed in Section "Managerial insights (step five)".
• Our interview sample may have been biased. We tried to create a diverse sample of interviewees from various European countries. However, many interviewees were located in Germany (*N* = 13), probably because all the authors are German. This may have led to biases in the answers, as transport systems differ at a country level. Such biases must be acknowledged as part of the research process. However, we expected that the interviewees would have the necessary expertise to answer our questions from a broader European point of view, excluding their own biases.
• Transcribing interviews is time-consuming but very important. We encourage researchers to carry out the transcriptions themselves, as this helps in-depth analysis of the interview data. Notes and memory protocols only save fractions of the content. As our analysis of the interview transcripts using Microsoft Excel was very time-consuming, programs that are more practical for analyzing large amounts of textual data are recommended, such as Atlas.ti or NVivo. Text-mining techniques, such as topic modeling or building a document-term matrix (DTM), might also be leveraged at this stage to analyze the interview transcripts systematically.
• Interviews might have been considered for the first Delphi round. As described above, we had too many interviewee dropouts after the first round with the Delphi questionnaire, and therefore decided against this.
• The framing and tense of the Delphi questionnaires could have been improved. All questions should be formulated positively or negatively, and if these are mixed, the questions should be recoded in the data analysis. We formulated all projections in the future tense; however, the present tense may be more suitable, especially when the time horizon is provided. We encourage researchers to check these issues before distributing their Delphi questionnaires.
• The actual time needed to fill in the Delphi questionnaire may vary. We received mixed feedback from the panelists on the number of projections. Although pre-tested several times, some panelists considered the questionnaire to be too long and time-consuming. One panelist canceled the process as it took too much time. We encourage researchers to keep their questionnaires as short as possible. However, this may conflict with collecting sufficient amounts of data. To avoid long questionnaires, it may also be advisable to define the scope of the study clearly, and to clarify any basic assumptions beforehand.
• Alternative questionnaire formats may have advantages for researchers. Analyzing a Word document, transferring the results into table format for analysis, and preparing the second round personalized for more than 40 panelists can be very time-consuming and prone to errors. As an alternative questionnaire format, we recommend considering the real-time Delphi (RTD) format [18] (using a platform) and/or sending an url. The RTD also has other advantages, such as immediate feedback. Examples of the use of RTD in mobility research include Julsrud and Uteng [24] and Spickermann et al. [48]. However, such platforms may be costly.

## Selection of panelists (step two)

Before approaching panelists for our Delphi study, pre-selection criteria were defined. Panelists should come from diverse backgrounds to mitigate biases [7]. As we focused on D2D air travel, we tried to capture this scope through an appropriate selection of panelists (see also Section "Expert interviews"). We included panelists working not only in aviation, but also at airports, as ground-transport providers (providing mobility transport to and from airports), and in the broader mobility sector. Ten stakeholder groups were identified for the panel, representing all main travel segments. As all subgroups were part of the mobility industry more broadly, the Delphi exercise was still conducted with a single industry panel [16]. Additional selection criteria were a minimum of two years’ work experience in the mobility industry, and a European working scope. All panelists were anonymized in the results.

**Fig. 3:**
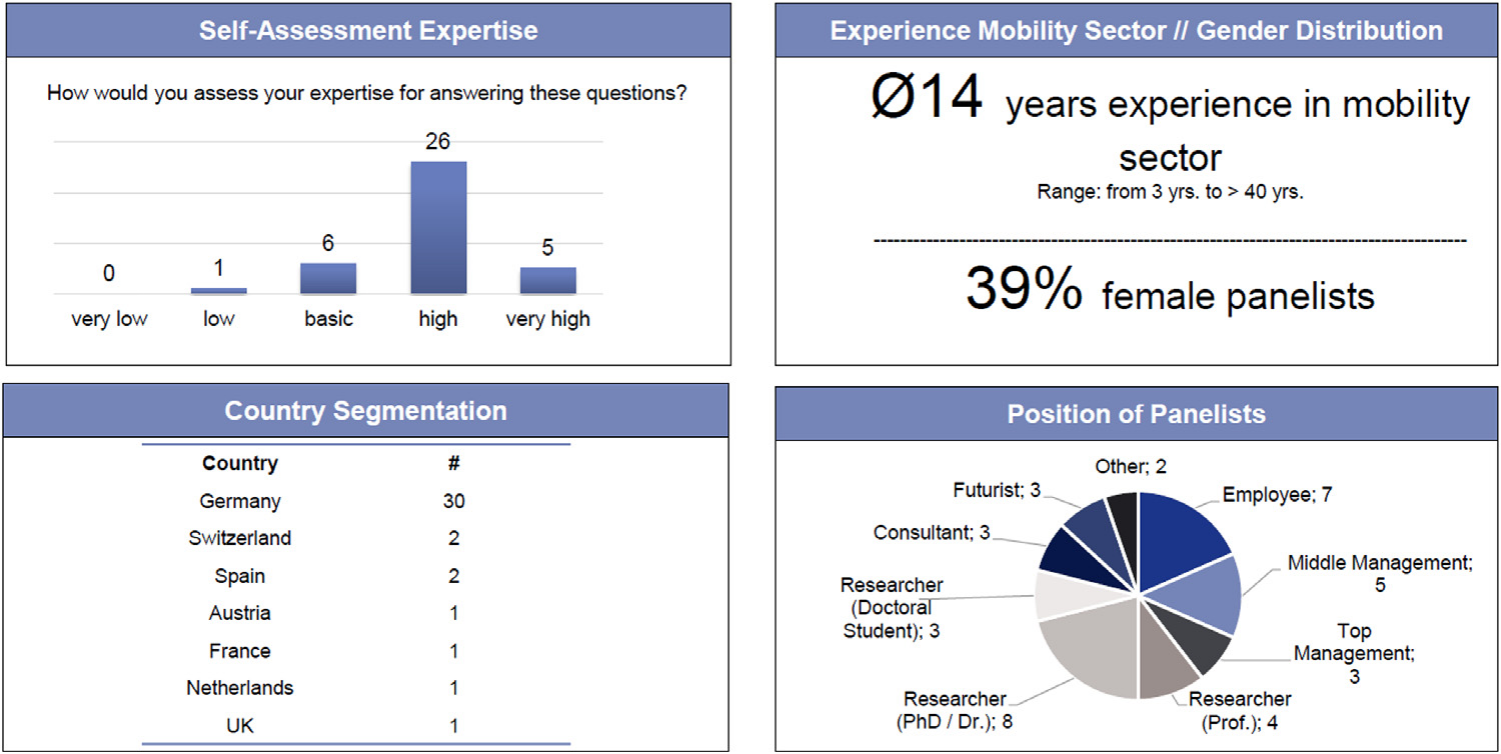
Overview of final panelists [26].

Potential panelists were identified by leveraging our personal networks, scanning conference lists and searching LinkedIn. All contacts were tabulated, with information on name, country, position, contact details, whether or not they returned the questionnaire in rounds one and two, and general comments. Each panelist was assigned an ID,^4^ starting at 1. As the panelists’ overviews contained personal information, they had to be stored with extra care, accessible only to the researchers. A total of 113 people were contacted via email, 16 of whom declined the request, mainly due to personal time constraints, extended absence or self-assessing themselves as having insufficient expertise. Eight panelists who agreed to participate either did not return their questionnaires at all, or submitted responses with too many missing values. Forty-one people did not respond, and five people could not be contacted owing to incorrect email addresses. In total, 43 panelists participated in the first round, leading to a response rate of 38%.^5^ As five of these dropped out in the second round, the overall response rate was 34%. Fig. 3 provides an overview of the final panel. Thirty-one panelists assessed their expertise for assessing the questions as “high” or “very high”. Hence, we assumed a valid selection process and suitable panelists for our Delphi study. The panel was also diverse in terms of job position and gender distribution. As this study was conducted in the male-dominated mobility sector, we considered 39% female panelists to be a good result.

### Lessons learned from selecting the panelists (step two)

• Biases might occur at any step of the research process and should be acknowledged. As already discussed, leveraging personal networks to acquire panelists may create bias [35]. Fig. 3 shows that our panel was German-heavy, as previously identified in Section "Expert interviews". Additionally, panelists might have been more involved due to personal connections with the research team. Conversely, selecting a diverse panel (different age, gender, positions, companies etc.) is a strategy to mitigate biases [7].
• We focused in our panel selection on the “*surface-level variables*”, such as age, gender, or job position [49]. Additionally, we recommend exploring “*deep-level diversity criteria*”, such as personal values or beliefs, as explored in an experiment by Spickermann, Zimmermann, & von der Gracht [49]. Deep-level criteria can potentially affect the panel diversity and response behavior of participants. Conversely, it might be difficult to explore deep-level criteria and require further questions and / or prior knowledge of the panelists. Mauksch et al. [35] provides a recent review of tools for the selection of panelists.
• Online social networks are suitable channels for researching and selecting panelists. LinkedIn provides sufficient information to decide whether someone is knowledgeable and might be considered to be an expert (based on work experience, professional field, etc.). Depending on the study's scope and research question, other online social networks might be considered as alternative sources, such as Instagram, Facebook or industry-focused networks (including conference lists, also partly used in our study).
• Selecting and approaching panelists generates personal data, including names, levels of expertise and email addresses, as well as private information such as times of absence (e.g. through receiving out-of-office replies). We advise researchers to be extremely careful with such data, even after the study has been conducted. Data must be stored securely at all times, including when projects have ended and papers have been published. There are several ways to store private data, for example through password protection. Personal data may also be deleted after some time, although this should be checked against the terms and conditions of project partners, employees, universities, publishers, etc.
• After executing the Delphi study, we only contacted the panelists to distribute the results. The panelists agreed to take part in the Delphi study, but not to receive newsletters, invitations or other non-study-related content. Only two panelists explicitly contacted us afterwards to “keep in touch”.

## Execution of the Delphi study (step three)

We approached the panelists via a short email message, which introduced our study and specified the deadline for return. The questionnaire was attached as a Word document in read-only format.^6^ We allowed a minimum of two weeks for panelists to return their questionnaires. We sent email reminders, and telephoned the panelists to ask for their assessments. Delphi rounds can be repeated until consensus among panelists is reached. In our case, two rounds were sufficient. We were also interested in projections that created dissent among the panelists. These diverging assessments were interpreted as uncertainty among the panelists with respect to particular projections. These findings provided interesting insights and were discussed separately in light of the current literature. We also wanted to avoid the risk of a high dropout rate in a potential third round.

In interim analysis between rounds one and two, we checked the raw data for any errors (missing values or double answers). When errors were identified, we reached out to the panelists and requested corrected estimations. This enabled us to include as many questionnaires as possible. We received 504 qualitative comments in the first round, which might have been overwhelming. Similarly to Schuckmann et al. [47], we divided all the comments into two subgroups containing supporting or non-supporting arguments. This was carried out in Microsoft Excel using a simple table format. For the quantitative analysis, we conducted descriptive statistics in R and calculated the mean, median, standard deviation and interquartile range (IQR). R, a statistical software package, is suitable for analyzing Delphi data as it is open-source and free, easy to use (even for beginners), replicable with the use of codes, and based on packages, allowing continuous improvement and hands-on analysis. Among other measurements, the IQR (Q3 – Q1) is a common consensus measurement widely used in applied Delphi studies, as highlighted in von der Gracht's [55] literature review. It depicts 50% of assessments between the lower quartile (Q1) and upper quartile (Q3). We applied a threshold of IQR ≤ 1 to detect which projects gained consensus. These were used for the scenario development at a later research stage. Projections with the highest IQR attracted the most divergent opinions and were discussed separately, providing further interesting insights. No data standardization was necessary, as the same (7-point Likert) scale was used for all answers. Boxplots provided a quick and easy visual summary of the data, and allowed outliers to be identified. Scatter plots provided a first indication of possible clusters. Although we used an ordinal scale to assess the projections, we conducted descriptive statistics to reflect on the spread of answers among panelists. The standard deviation and the change in standard deviation (in%) between the first and second rounds were interpreted as indications of the level of convergence in panelists’ assessments. A convergence was also supported by panelists’ comments in the second round. Histograms of the mean, minimum and maximum depicted the distribution of answers at a quick glance.

The second-round questionnaire was again constructed in Microsoft Word. Panelists received controlled and personalized feedback in the form of the spread of the aggregated group response in histograms, including their own estimations, and a summary of the rationale for the group response (taken from panelists’ comments). An example of projection one is given in Fig. 7 in the supplementary material. We also sent them feedback on all the projections, including those that had already reached consent in the first round. In light of Delphi's characteristics, the panelists had an opportunity to compare their own estimations and to reconsider their answers based on the group response of the entire panel. Having made their assessments, they returned their questionnaires by email to the researchers.

### Lessons learned from executing the Delphi study (step three)

• Conducting a Delphi study is a slow process. Receiving feedback and returns may take several weeks, so a long period for data gathering must be considered in planning projects. Hence, we advise researchers to plan a suitable back-up period in case returns are slower than expected. It is also advisable to return the controlled feedback in the second round as soon as possible to maintain momentum. Conversely, the required time might also depend on the sample size, type of experts, access to experts, and researchers’ networks.
• Timing is important. To avoid a low response rate, the questionnaire distribution should not overlap with holiday periods, such as summer break or Christmas. Spring and autumn are ideal seasons for data gathering.
• Qualitative data in Delphi studies can be analyzed in systematic ways. Text-analysis software can be used to support the execution of a Delphi study. Our study required analysis of a large amount of qualitative textual data (panelists’ comments). As previously discussed, investment in a software package for qualitative data analysis may support the research process. Roßmann et al. [42] applied a content analysis approach and coded the qualitative comments by two human coders using pre-defined units. Applying grounded theory, von Briel [53] derived core statements by coding panelists’ responses to open-ended questions from a first Delphi round. These core statements are used as input for the second Delphi round.
• Various consensus measurements are possible and should be applied. We used the IQR because it is widely used in applied Delphi studies. However, other statistical indices are available for measuring consensus, agreement and association [37]. Dajani, Sincoff, and Talley [11] provide termination (or stopping) criteria for Delphi rounds by testing the stability with the x^2^-test. The level of agreement in our data might have differed if we had chosen different indices. In retrospect, we should have used more indices to test our data, the stability and to compare results.
• We consider projections that did not receive consent among the panelists as valuable findings. Although not used for further scenario development, we believe that these projections can be discussed as developments that lead to high uncertainty.

## Development of scenarios (step four)

The scenario development approach on its own is already well established as a suitable foresight method in many disciplines [52]. As shown by Nowack, Endrikat, & Guenther [40], many Delphi studies are used for scenario building. Scenarios help to shed light on what the future might look like. They help researchers to communicate findings in a descriptive, easily understandable way for readers and practitioners. To create scenarios for the possible future of D2D air travel, we conducted a cluster analysis in R, using data on seven projections that reached the IQR threshold. For this, we used the means of the assessed probability, impact and desirability of each projection. A detected cluster was considered as a scenario on its own. Various algorithms were available, and the results of different cluster approaches were compared to determine the most feasible results. We applied fuzzy c-means clustering (FCM) [6], k-means clustering [32], partitioning around medoids (PAM clustering) [46] and hierarchical clustering (HC) [25], [39]. Prior Delphi studies applied mainly fuzzy clustering (e.g. [17], [42]) and HC (e.g. [31], [50]). To detect the optimal number of clusters (required for some algorithms but not needed for HC), we applied Charrad et al.'s [10] majority rule using the *NbClust* package.

With seven confirmed projects, our data set was rather small. A higher IQR-threshold might have increased the data set. According to von der Gracht [55], studies with more answer options (e.g. a 10-point Likert scale) may enlarge the IQR-threshold. However, this did not apply to our study. A scatter plot provided a first visual indication of possible clusters. The research team compared the results of all cluster algorithms described above and selected results that provided meaningful clusters. Fuzzy, k-means and HC clustering generated the same clusters. We decided on HC, as Akman, Comar, Hrozencik, and Gonzales [2] argue that it is a suitable cluster algorithm for a small data set. Hence, hierarchical clustering with Euclidean distance and using the Ward [57] method provided the best and most feasible results for our study. Our R code is attached in the supplementary material.

**Fig. 4:**
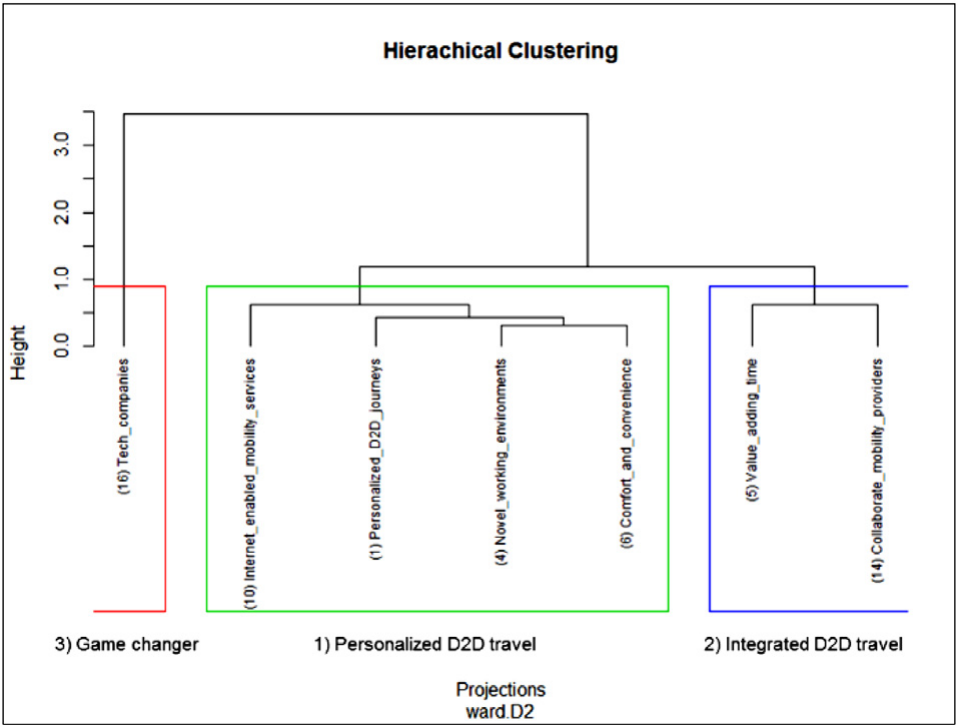
Dendrogram of three future scenarios in nested structure for D2D mobility in 2035.

We developed three clusters leading to three separate scenarios: 1) personalized D2D travel, 2) integrated D2D travel, and 3) the game changer. The average probability, impact and desirability of each developed scenario are depicted in Table 1. A dendrogram displaying the three cluster assignments in the nodes is presented in Fig. 4. To describe the scenarios, we mainly utilized panelists’ comments from the two Delphi rounds, including supporting and non-supporting arguments. Findings from the literature review and the expert interviews in step one supported the development when appropriate. For instance, an expert might provide a good description of a projection concerning the value-added use of travel time. Scenario 1 (personalized D2D travel) and scenario 2 (integrated D2D travel) are not mutually exclusive but have different foci. Scenario 1 is concerned with high personalization of journeys, whereas scenario 2 focuses on partnerships. Scenario 3 (the game changer) is a black swan scenario: if it were to become reality, it would have the potential to seriously disrupt the aviation and travel industry. All three scenarios depict a possible future. However, some trends, like digitalization and personalization, relate to all three scenarios. Other scenario studies might develop scenarios that are completely independent of each other. We argue that this depends on the research scope and the overall research question.

**Table 1:** Cluster statistics for results from HC (example from [27]).

Scenario name	Included projections	Probability *(mean)*	Impact *(mean)*	Desirability *(mean)*
1) Personalized D2D travel	1, 4, 6,10	6.04	5.81	5.46
2) Integrated D2D travel	5, 14	6.29	6.08	5.97
3) Game changer	16	4.53	5.11	4.18

### Lessons learned from developing scenarios (step four)

• Scenarios help to present research results in a vivid manner and easily to communicate for readers and practitioners. This applies not only to Delphi studies, but to all research incorporating scenario development.
• Applying a cluster analysis provides a systematic and transparent way to build scenarios. It may also exclude the subjective view of the research team, however, the final decision on the clusters and interpretation often follows a more heuristic approach [50]. Next to the Delphi-based scenario approach described in this paper, de-bias strategies are also available in this broader context of scenario development in an overview by Schirrmeister et al. [45].
• Choosing a cluster algorithm and number of clusters is not easy. With regard to the chosen algorithm, Charrad et al.’s [10] majority rule may provide support. The R package applies 30 clustering validity indices simultaneously, and makes recommendations based on the number of clusters most frequently mentioned among all indices. This can save time and provides a rigorous basis for cluster analysis and ensuing scenario development.
• Scenarios should be named and described. They might, of course, simply be called 1) probable future developments, 2) possible future developments and 3) alternative future. During the Delphi study, we received the feedback to select individual names to make our results appealing and clear. As described above, comments by panelists may help to describe scenarios. The literature review and expert interviews may also provide support.

## Managerial insights (step five)

Scenarios can support organizations in the pre-planning phase, shedding light on the possible future environment of their businesses [44]. Deriving managerial insights from final Delphi study results can make them impactful and relevant to practitioners and the industry. We derived managerial implications for different stakeholder groups from our panel, and customized our results for airports, airlines and public transport providers throughout the D2D air travel value chain. In other words, we sought to determine *how the results and developed scenarios would affect the mobility sector and organizations.* As we consider the derivation of managerial insights to be essential, we included this in our research approach.

**Fig. 5:**
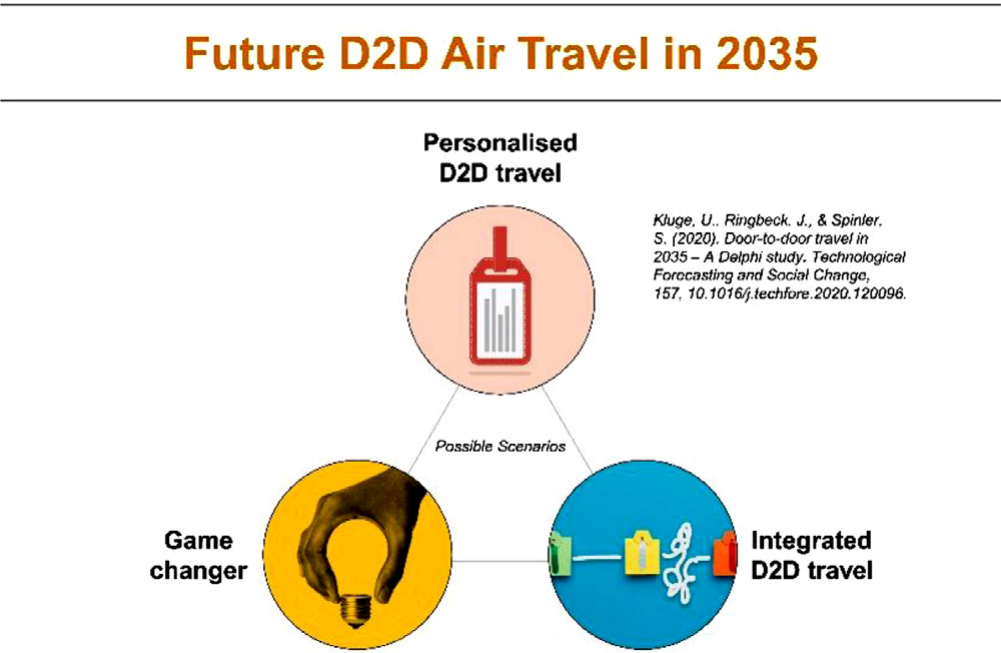
Example of the visualization of scenarios [4].

Incentives may be helpful for recruiting panelists, such as social recognition (name people on the panel list), providing a copy of the final research results or offering vouchers.^7^ We found that distributing the results was a sufficient incentive for our study. Hence, we prepared a summary of our Delphi study results and distributed this report to all panelists [26]. The summary was short but comprehensive, with references to the original publication. The panelists had professional, mobility-related backgrounds, and their replies indeed confirmed that our study results were useful for their personal work. To present the developed scenarios in a more practical way, we also considered it useful to work with visualizations, which made it easier to understand and represent the scenarios, as depicted in Fig. 5.

### Lessons learned from managerial insights (step five)

• Deriving managerial insights highlights the practical relevance of the Delphi study results. To increase their relevance, we tailored our managerial insights to the main stakeholder groups in our study. For instance, through the lens of airlines, how would our Delphi study results affect airline businesses today and in 17 years’ time?
• Presentation is important. We learned that presenting our results in a visualization or descriptive figure helped to disseminate our findings beyond the scientific community. Fig. 5 depicts an example.
• Providing a summary of the Delphi study findings may be an incentive for recruiting panelists. Researchers are advised to prepare a summary and distribute it on completion of the study. Alternatively, the published paper or report may be distributed, although panelists may have little time to appreciate a comprehensive report.

### Method validation

We regard research methods like the Delphi technique as appropriate for investigating novel topics such as future D2D air travel. The results provide initial insights into possible future developments that would otherwise be difficult to identify. Until the future has become the present, it is impossible to obtain any quantitative data to validate such research. Hence, we highly recommend conducting an a priori literature review to provide a theoretical framework for the development of projections and to increase the rigor of the research. Triangulation of methods can be ensured by using several methods in developing projections.

Our results are highly relevant to both academia and management practice, for example in enabling identification of emerging research areas and supporting the pre-planning process within organizations. However, we do not consider our research results to be useful for enabling theory building. Hence, we believe that further research is necessary for that purpose. As explored in Section "Method details and co-submitted research", the Delphi technique can also be used to address other research questions and current issues, where validation steps might apply. In such context, Delphi studies might be more useful for theory building. However, this is not the scope of this paper.

## Reflection and conclusion

### Reflection on the method

Although the Delphi technique can be time-consuming, requires much effort, and can be costly, we consider the technique as valuable for several reasons: (1) it allowed us to incorporate the desired D2D air travel, multi-stakeholder scope in the research, (2) we were able to include industry expert knowledge in our study that aims to answer a practical research question and is hence of high relevance for the industry side, (3) the technique can be combined with other methods, such as with interviews, workshops, scenario development and other methods (as all described above), and (4) we believe it is a universally applicable technique, suitable for students, early-career researchers, and more experience researchers across all disciplines.

Overall, we believe that our Delphi study provides valuable results for the industry by deriving managerial insights at the end of the process. Additionally, the new scope of D2D air travel provides valuable contributions for the academia and policy makers. Recent EU-funded projects focus on intermodal air travel [9], [13], thus showing the high practical relevance of this scope. There are, however, some limitations applying the technique. We developed three possible future scenarios but since a few months, the Corona-related crisis is affecting the travel and aviation industry heavily. Passengers’ demand for air travel dropped for European carriers more than 80% (September 2020 vs. 2019) [22]. Nobody can foresee how this crisis will change the aviation industry and D2D travel chains in the long term. We did not include such development in our study as we conducted the research in 2018 with no information on that matter. It could have been included as a game changer scenario; however, one can conclude that there are uncertainties that the Delphi technique cannot capture. We live in an ever-changing environment with technology, society, policies, laws, and other areas changing at a fast pace. One always needs to reflect on the context in which panelists provide their assessments in the Delphi study.

### Conclusion

In this technical paper, we provide a replicable five-step research process for conducting a two-round Delphi-based scenario study examining the future of D2D air travel in 2035. Lessons learned from applying this framework are provided, including advice for other researchers. We consider the Delphi technique to be a valuable research tool that can be applied in many disciplines and areas of research. It is a technique for answering prospective research questions, but can also be used for other research purposes. Although applied mainly in the fields of healthcare, education and business research, we encourage researchers from other fields to apply the Delphi method.

## Acknowledgments

Parts of the Delphi study were conducted within the CAMERA-project. CAMERA has received funding from the European Union's Horizon 2020 Research and Innovation Program under grant agreement No. 769606.

## Declaration of competing interest

The authors declare that they have no known competing financial interests or personal relationships that could have appeared to influence the work reported in this paper.

## Appendix Supplementary materials

Image, application 1

## Footnotes

1 In the co-submitted research paper [27], we refer to a four-step approach and use the term “pre-study” in the context of step 1, which is not used here. In this technical paper, the research steps are presented in more detail.

2 Other Delphi studies conducted between 14 and 18 interviews [14], [23], [51]. Hence, we considered 18 expert interviews for our Delphi study as sufficient and a valid number in line with the literature.

3 Seventeen of the 18 Interviews were conducted as part of CAMERA, an EU-funded project (GA 769606, H2020).

4 The IDs were used in the research process to save personalized questionnaires in the second Delphi round, to maintain the anonymity of returns, and to work with the data set. To detect the panelists’ names, it was necessary to access the panelists’ overview table, which was stored securely.

5 Similar offline (vs. RT Delphi) and two-round Delphi studies show a response rate of 43% and 42% [34], [56]. Hence, we considered our response rate as sufficient.

6 Read-only formats still allow questionnaires to be filled in.

7 Use of vouchers may introduce limitations or biases and must be discussed at the planning stage.
