# Supplementary material for: Lessons Learned from a Two-Round Delphi-based Scenario Study
Source: MethodsX. 2020 Dec 13;8:101179. doi: 10.1016/j.mex.2020.101179 (PMC7749424; doi:10.1016/j.mex.2020.101179)
Supplement: Supplementary file 1 [file mmc1.docx]

# SUPPLEMENTARY MATERIAL

**Table 2:** Overview top five cited papers across disciplines (Scopus search with keyword “Delphi” November 2020)

| Discipline | Author(s) | Year | Journal | Title | Cited by |
| --- | --- | --- | --- | --- | --- |
| Healthcare* | Singer et al. | 2016 | *JAMA - Journal of the American Medical Association* | The third international consensus definitions for sepsis and septic shock (sepsis-3) | 6063 |
|  | Stang | 2010 | *European Journal of Epidemiology* | Critical evaluation of the Newcastle-Ottawa scale for the assessment of the quality of nonrandomized studies in meta-analyses | 5420 |
|  | Cummings et al. | 1994 | *Neurology* | The neuropsychiatric inventory: Comprehensive assessment of psychopathology in dementia | 5294 |
|  | Palisano et al. | 1997 | *Developmental Medicine and Child Neurology* | Development and reliability of a system to classify gross motor function in children with cerebral palsy | 3893 |
|  | Dellinger et al. | 2008 | *Critical Care Medicine* | Surviving sepsis campaign: International guidelines for management of severe sepsis and septic shock: 2008 | 3743 |
| Engineering | Zhang et al. | 2008 | *Osteoarthritis and Cartilage* | OARSI recommendations for the management of hip and knee osteoarthritis, Part II: OARSI evidence-based, expert consensus guidelines | 1813 |
|  | McAlindon et al. | 2014 | *Osteoarthritis and Cartilage* | OARSI guidelines for the non-surgical management of knee osteoarthritis | 1440 |
|  | Schael et al. | 2006 | *European Physical Journal C* | Search for neutral MSSM Higgs bosons at LEP | 581 |
|  | Zhang et al. | 2007 | *Osteoarthritis and Cartilage* | OARSI recommendations for the management of hip and knee osteoarthritis, Part I: Critical appraisal of existing treatment guidelines and systematic review of current research evidence | 565 |
|  | Achanga et al. | 2006 | *Journal of Manufacturing Technology Management* | Critical success factors for lean implementation within SMEs | 546 |
| Social Science | Hsu & Sandford | 2007 | *Practical Assessment, Research and Evaluation* | The Delphi technique: Making sense of consensus | 1965 |
|  | Brooks, Adger, & Kelly | 2005 | *Global Environmental Change* | The determinants of vulnerability and adaptive capacity at the national level and the implications for adaptation | 996 |
|  | Deardorff | 2006 | *Journal of Studies in International Education* | Identification and assessment of intercultural competence as a student outcome of internationalization | 734 |
|  | Osborne et al. | 2003 | *Journal of Research in Science Teaching* | What "ideas-about-science" should be taught in school science? A delphi study of the expert community | 515 |
|  | Choi & Sirakaya | 2006 | *Tourism Management* | Sustainability indicators for managing community tourism | 440 |
| Business Research** | Okoli & Pawlowski | 2004 | *Information and Management* | The Delphi method as a research tool: An example, design considerations and applications | 1687 |
|  | Rowe & Wright | 1999 | *International Journal of Forecasting* | The Delphi technique as a forecasting tool: Issues and analysis | 1108 |
|  | Schmidt et al. | 2001 | *Journal of Management Information Systems* | Identifying software project risks: An international Delphi study | 769 |
|  | Landeta | 2006 | *Technological Forecasting & Social Change* | Current validity of the Delphi method in social sciences | 567 |
|  | Gartner | 1990 | *Journal of Business Venturing* | What are we talking about when we talk about entrepreneurship? | 559 |
| Computer Science | Schmidt et al. | 2001 | *Journal of Management Information Systems* | Identifying software project risks: An international Delphi study | 769 |
|  | Lee et al. | 2009 | *Expert Systems with Applications* | A green supplier selection model for high-tech industry | 477 |
|  | Brancheau & Wetherbe | 1987 | *MIS Quarterly: Management Information Systems* | Key issues in information systems management | 475 |
|  | Brancheau, Janz, & Wetherbe | 1996 | *MIS Quarterly: Management Information Systems* | Key issues in information systems management: 1994-95 SIM delphi results | 470 |
|  | Niederman, Brancheau, & Wetherbe | 1991 | *MIS Quarterly: Management Information Systems* | Information systems management issues for the 1990s | 452 |
| Environmental Science | Brooks et al. | 2005 | *Global Environmental Change* | The determinants of vulnerability and adaptive capacity at the national level and the implications for adaptation | 996 |
|  | Landrigan et al. | 2002 | *Environmental Health Perspectives* | Environmental pollutants and disease in American children: Estimates of morbidity, mortality, and costs for lead poisoning, asthma, cancer, and developmental disabilities | 339 |
|  | Kannan et al. | 2002 | *Environmental Science and Technology* | Perfluorooctanesulfonate and related fluorinated hydrocarbons in marine mammals, fishes, and birds from coasts of the Baltic and the Mediterranean Seas | 336 |
|  | Seuring & Müller | 2008 | *Business Strategy and the Environment* | Core issues in sustainable supply chain management - A Delphi study | 316 |
|  | James et al. | 2009 | *Urban Forestry and Urban Greening* | Towards an integrated understanding of green space in the European built environment | 236 |
| Physics & Astronomy | Amaldi, de Boer, & Fürstenau | 1991 | *Physics Letters B* | Comparison of grand unified theories with electroweak and strong coupling constants measured at LEP | 1218 |
|  | Heister et al. | 2003 | *Physics Letters, Section B: Nuclear, Elementary Particle and High-Energy Physics* | Search for the standard model higgs boson at LEP | 989 |
|  | Bagger et al. | 2006 | *Physics Reports* | Precision electroweak measurements on the Z resonance | 981 |
|  | Schael et al. | 2006 | *European Physical Journal C* | Search for neutral MSSM Higgs bosons at LEP | 581 |
|  | Abreu et al. | 1996 | *Nuclear Instruments and Methods in Physics Research, Section A: Accelerators, Spectrometers, Detectors and Associated Equipment* | Performance of the DELPHI detector | 309 |
| Biochemistry, Genetics & Molecular Biology | Jordan et al. | 2003 | *Annals of the Rheumatic Diseases* | EULAR Recommendations 2003: An evidence based approach to the management of knee osteoarthritis: Report of a Task Force of the Standing Committee for International Clinical Studies Including Therapeutic Trials (ESCISIT) | 1468 |
|  | Smolen et al. | 2010 | *Annals of the Rheumatic Diseases* | Treating rheumatoid arthritis to target: Recommendations of an international task force | 1355 |
|  | Smolen, Landewé, et al. | 2010 | *Annals of the Rheumatic Diseases* | EULAR recommendations for the management of rheumatoid arthritis with synthetic and biological disease-modifying antirheumatic drugs | 1233 |
|  | O’mahony et al. | 2015 | *Age and Ageing* | STOPP/START criteria for potentially inappropriate prescribing in older people: Version 2 | 903 |
|  | Zhang et al. | 2006 | *Annals of the Rheumatic Diseases* | EULAR evidence based recommendations for gout. Part II: Management. Report of a task force of the EULAR Standing Committee for International Clinical Studies Including Therapeutics (ESCISIT) | 901 |

**Healthcare includes medicine, nursing and health professions according to Scopus subject areas*

***Business research includes business, management, and accounting according to Scopus subject areas*

**Table 3:** Overview lessons learned and technical publications on the Delphi technique

| Author(s) | Year | Journal | Title |
| --- | --- | --- | --- |
| Bolger & Wright | 2011 | *Technological Forecasting & Social Change* | Improving the Delphi process: Lessons from social psychological research |
| Frewer et al. | 2011 | *Technological Forecasting & Social Change* | The use of Delphi methodology in agrifood policy development: some lessons learned |
| Goluchowicz & Blind | 2011 | *Technological Forecasting & Social Change* | Identification of future fields of standardisation: An explorative application of the Delphi methodology |
| Hasson & Keeney | 2011 | *Technological Forecasting & Social Change* | Enhancing rigour in the Delphi technique research |
| Hussler, Muller, & Rondé | 2011 | *Technological Forecasting & Social Change* | Is diversity in Delphi panelist groups useful? Evidence from a French forecasting exercise on the future of nuclear energy |
| Nowack, Endrikat, & Guenther | 2011 | *Technological Forecasting & Social Change* | Review of Delphi-based scenario studies: Quality and design considerations |
| Zimmermann, Darkow, & von der Gracht | 2012 | *Technological Forecasting & Social Change* | Integrating Delphi and participatory backcasting in pursuit of trustworthiness - the case of electric mobility in Germany |
| Förster & von der Gracht | 2014 | *Technological Forecasting & Social Change* | Assessing Delphi panel composition for strategic foresight – A comparison of panels based on company-internal and external participants |
| Gray & Morris | 2016 | *Issues in Information Systems* | The Delphi Technique: Lessons Learned from a first time researcher |
| Jünger et al. | 2017 | *Palliative Medicine* | Guidance on Conducting and REporting DElphi Studies (CREDES) in palliative care: Recommendations based on a methodological systematic review |
| Hirschhorn | 2019 | *International Journal of Social Research Methodology* | Reflections on the application of the Delphi method: lessons from a case in public transport research |
| Belton et al. | 2019 | *Technological Forecasting & Social Change* | Improving the practical application of the Delphi method in group-based judgment: A six-step prescription for a well-founded and defensible process |
| Markmann et al. | 2020 | *Futures & Foresight Science* | Improving the question formulation in Delphi‐like surveys: Analysis of the effects of abstract language and amount of information on response behavior |
| Mauksch et al. | 2020 | *Technological Forecasting & Social Change* | Who is an expert for foresight? A review of identification methods |

# INTERVIEW GUIDE: EXPERT INTERVIEWS ON THE FUTURE OF MOBILITY

*The three main objectives of the interviews were:*

1. Characteristics of current & future passengers
2. Insights into methods used to understand the mobility market
3. The future of mobility: ways to travel, challenges, future modes of transport, need for action and investments

*The questions below were used as guiding questions:*

1. **Generals**

- Introduction (project, data protection, aim of interview)
- Company name and department/division of interviewee & area of expertise of interviewee

1. **Characterizing current and future customer markets**

- Why is it important to have knowledge about passengers?
- What are expectations, needs and requirements of customers/ passengers?
- Are any regulations, local or national government planning (current and future) might affect expectations, needs and requirements of mobility passengers?
- What methodologies are commonly used in the mobility sector to gain information about passengers (such as demographic information, travel or booking behavior)?
- How can we gain information about how passenger types, booking and travelling behavior is going to change?
- What are challenges or drawbacks when carrying out research on passengers?

1. **Future of mobility**

- How are we going to travel in the future (in 2035 and 2050)? What are the modes of the future?
- What are major challenges facing the overall transport system (e.g. ageing population, digitalization)?
- Are there any effects on current business models for the future?
- What are the jobs expected to emerge, change or disappear in the future transport workforce?
- Where is further research, action or investment necessary for improvement of the European transport system?

1. **Wrap up**

- Is there anything you would like to add at this point, which has not been addressed here but you consider as essential when thinking about the topics above?

**
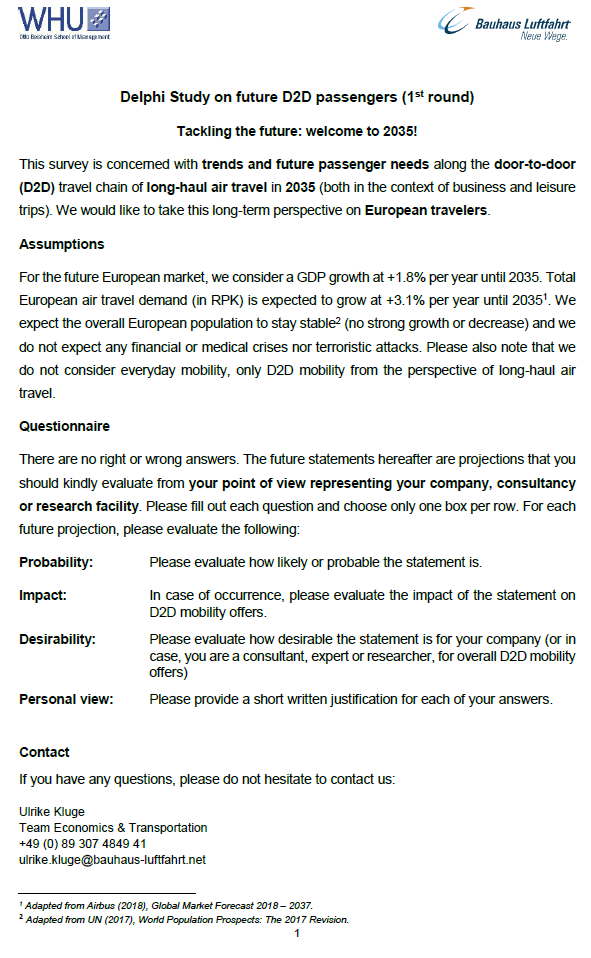
**

**
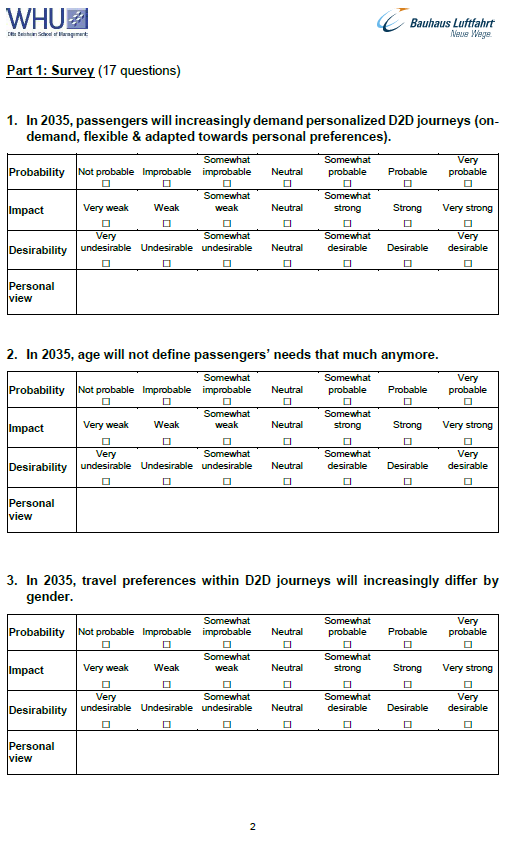
**

**
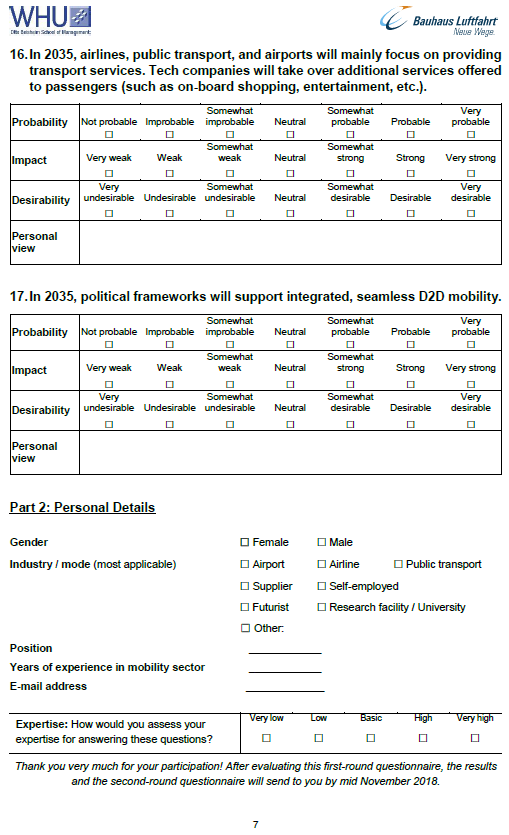
**

**Fig. 6:** Extract of Delphi questionnaire used in first round (p. 3-6 missing)


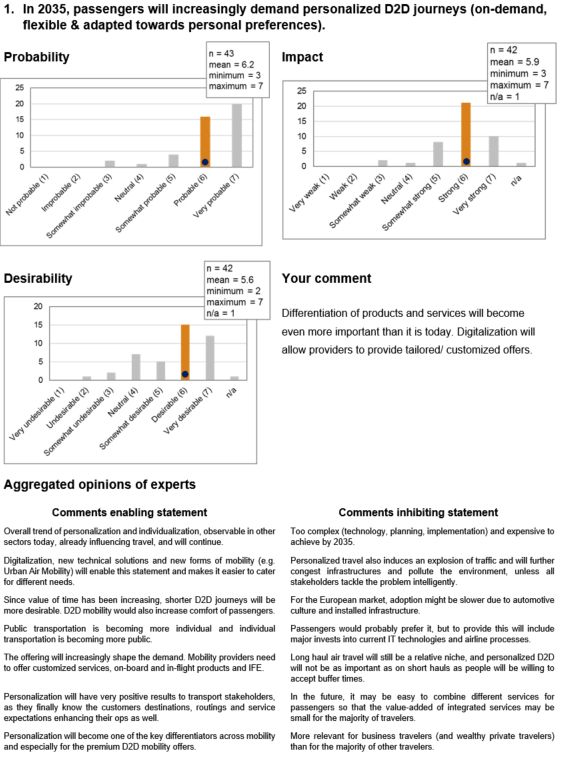


**Fig. 7:** Extract of Delphi questionnaire used in second round (for projection 1)

**CODE FOR HIERARCHICAL CLUSTERING in R**

# Required packages

require(readxl)

require(stats)

require(base)

require(graphics)

# upload data into R

## Create subset for clustering and look at structure

DataClust = data_file

head(DataClust)

## Hierarchical clustering (HC) using Ward's method. Two ward clustering; only ward.D2 uses ward (1963) hence, this is used for the Delphi study (but cluster results are both same in our study)

## Compute distance matrix

Delphi_distances <- dist(DataClust, method="euclidean")

## Conduct HC using ward's method (ward.D2)

## Set seed to make your results replicable

set.seed(42)

res.hc <- hclust(Delphi_distances, method="ward.D2")

res.hc$centers

str(res.hc)

## Cut tree into 3 groups

grp <- cutree(res.hc, k = 3)

## Visualize in dendrogram

plot(res.hc, cex = 0.75, ylab = "Height", xlab = "Projections", labels = DataClust$Consensus, main = "Hierachical Clustering", sub = "ward.D2", hang = -0.9)

## Plot tree (if you want, save as picture)

rect.hclust(res.hc, k = 3, border = 2:5)

## In case you need help, use help() function

help("hclust")

help("dist")

help("cutree")

help("plot")
